# The psychiatric phenotype in triple X syndrome: New hypotheses illustrated in two cases

**DOI:** 10.3109/17518423.2012.655799

**Published:** 2012-05-14

**Authors:** Maarten Otter, Constance T. R. M. Schrander-Stumpel, Robert Didden, Leopold M. G. Curfs

**Affiliations:** 1 Department of Child & Adolescent Psychiatry, Eleos, Ede, The Netherlands; 2 Department of General Psychiatry, Eleos, Ede, The Netherlands; 3 Trajectum, Community Mental Health Team in ID, Zutphen, The Netherlands; 4 Department of clinical genetics, Maastricht University Medical Center UMC, Maastricht, The Netherlands; 5 GROW School for Oncology and Developmental Biology, Maastricht University Medical Center UMC, Maastricht, The Netherlands; 6 Behavioural Science Institute, Radboud University Nijmegen, Nijmegen, The Netherlands; 7 Department of Clinical Genetics, Maastricht University Medical Center UMC, Maastricht, The Netherlands

**Keywords:** Triple X syndrome, behavioural phenotype, development in adults, occupational development, sexual trauma, autism spectrum disorders

## Abstract

*Background:* Triple X syndrome (47,XXX or trisomy X) is a relatively frequent cytogenetic condition with a large variety of physical and behavioural phenotypes.

*Method:* Two adult patients with a triple X karyotype are described.

*Results:* Their karyotype was unknown until some years ago. What these patients have in common is that they were diagnosed with a broader autism phenotype, they were sexually abused, they suffer from psychotic illness and they show challenging behaviour, suicidality and a decline in occupational capacity.

*Discussion:* These gene-environment interactions are discussed. Gene-environment interactions may explain the variety of behavioural and psychiatric phenotypes in triple X syndrome. Ongoing atypical development in adults is hypothesized.

*Conclusions:* Gene-environment interactions and ongoing atypical development in adults should be taken into account in research concerning the psychiatric phenotype of developmental disorders, especially those involving triple X syndrome.

## Introduction

Since clinical genetic examination is recommended in developmental delay [1] and in autism spectrum disorders (ASD) [2], clinicians in the field of learning disabilities will more often be confronted with the results of clinical genetic examination. Triple X syndrome is a relatively frequent cytogenetic condition occurring in one in 1000 females [3], with a high variety of physical and behavioural phenotypes [4–6]. Most of the girls and women with an extra X chromosome develop without major developmental and intellectual disabilities. However, mental health problems have proven to be common in an unbiased population [7]. Full-scale intelligence quotient (IQ) scores clustered in the 85-90 range [7]. Psychotic features have long been described in women with triple X syndrome in biased populations, such as during cytogenetic screenings in asylums. Polani [8] calculated that a 47,XXX finding is 4-times more common in women with a psychotic disorder than in general. A study on autism, language and communication showed no ASD in girls with a 47,XXX karyotype [9].

The authors wish to share their experience with psychiatric conditions in two women with triple X syndrome by discussing various aspects of these findings.

## Method

Retrospective chart reviews were used to study development into adulthood in two women with triple X karyotypes. Psychological and psychiatric diagnoses of these two women were made by an experienced team of clinical psychologists and psychiatrists working in a residential facility for patients with a mild intellectual disability (ID), challenging behaviour and/or psychiatric disorders. Both patients enjoy sheltered living in this residential facility.

### Patient A

The patient was raised in a family with both parents and two siblings. Developmental milestones were reached on time. At 2 years of age the girl was hospitalized for dehydration and a viral infection. She reacted with nervous behaviour and complaints after coming home and continued to do so during childhood. She tended to wander about with strangers. After the third grade of primary school she switched to a school for special education. In secondary school she attended an agricultural college for children with special educational needs. The school physician referred her to a child and adolescent psychiatrist for the treatment of her odd behaviour, anxiety and acoustic hallucinations. She was sexually abused during her first psychiatric hospitalization when she was 17 years old. Thereafter, community mental healthcare and several periods of hospitalization were necessary to treat recurrent psychotic symptoms. She was readmitted to the facility where she still lives.

She is now 43 years of age. She lives in a group home for people with ID and severely challenging behaviour. Genetic diagnosis: mosaic 47,XXX (90%)/46,XX (10%) (2004).

Classification according to the Diagnostic and statistical manual of mental disorders-Fourth edition-Text Revision (DSM-IV-TR) [10]: axis I: Pervasive developmental disorder, not otherwise specified (PDD-NOS), multiple complex developmental disability (MCDD) type and post-traumatic stress disorder (PTSD). Axis II: mild ID level of functioning. Axis V: General Assessment of Functioning (GAF): challenging behaviour, extremely low self-esteem and mood instability requiring a highly structured programme. Instability occurs after changes in her living environment, like changes in the care-giving personnel, changes in the living group, etc. Deliberate self-harm, banging her head, swallowing glass and metal objects and suicidal behaviour occur in periods of instability. Reducing psychosocial stress is required to give her time to recover. In periods of stability she may enjoy visits and outings.

Psychological assessment (2007): Full-scale IQ (FSIQ) = 56, verbal IQ (VIQ) = 65, performance IQ (PIQ) PIQ = 53 (Wechsler Adult Intelligence Scale-Third edition-NederLands (WAIS-III-NL) [11]). Level of functioning equivalent to a toddler (Schaal voor emotionele ontwikkeling [12]; assessment of emotional development). High level of arousal, often complaining. Highly receptive to external and internal stimuli and impulsive reactions (Temperamentsschaal voor Zwakzinnigen (TVZ) [13]; assessment of temperament in ID).

To compare data from 15 years before: FSIQ = 73, VIQ = 74, PIQ = 78 (Wechsler Adult Intelligence Scale (WAIS) [14]). Level of emotional functioning was estimated to be equivalent to 7/8 years of age.

Current prescription: zuclopentixol decanoate, aripiprazole, carbamazepine and valproate as mood stabilizers, promethazine, biperiden, Depo-Provera, Movicol®, esomeprazole, simvastatin, metformin, levothyroxine 50 p.g daily. Medical history: Obesity, pulmonary embolism, diabetes mellitus type 2, hypothyroidism. No results of neuroimaging available.

### Patient B

The patient was the youngest girl in a family of four children. Developmental milestones were delayed. When she was 7 years old her parents divorced and she moved to a school for special education for children with mild learning difficulties. She later attended a secondary school for children with learning disabilities. As an adolescent she was sexually abused by her mother's boyfriend; she was consequently nervous and suffered from low self-esteem. She lived in a sheltered living environment in her adolescence and moved on to several other group homes due to behavioural disorders (deliberate self-harm and suicidal gestures). Several years of unskilled labour followed. Thereafter, she worked in sheltered workshops for mildly physically handicapped workers. She lost her job due to a ‘tennis elbow’ in both arms. After the breakup of an intimate relationship in 1996 she was hospitalized in an institute for the treatment of severe challenging behaviour in ID. She presented with a depressive illness with psychotic features, especially a tendency to paranoid cognitions and reactions with deliberate self-harm and suicidal gestures. She consequently stayed to live there.

She is 45 years of age. She lives in her own house in an apartment building for people with a mild ID and challenging behaviour. She visits a daytime activity centre in the same institute. Genetic diagnosis: 47,XXX (2004).

Classification according to DSM-IV-TR: axis I: PDD-NOS; post-traumatic stress disorder (PTSD) in remission. Axis II: mild mental retardation. Axis V: GAF: Adaptation problems in situations of mild psychosocial stress.

Psychological assessment (2005): FSIQ = 67; VIQ = 68; PIQ=67 (WAIS-III-NL). Visuo-motor impairments (The Bender Gestalt Test for young children [15]); attention deficits, concentration difficulties and low working speed (Bourdon-Vos test [16]); slightly impaired interference (Stroop test [17]); labile mood, easily frustrated, stubborn (Nederlandse Persoonlijkheids Vragenlijst (NPV) [18]; Dutch personality inventory; Nederlandse verkorte Minnesota Multiphasic Personality Inventory (MMPI) [19]; Dutch MMPI-short version; TVZ); PDD-NOS, no autism (Social Interpretation Test (SIT) [20]; Autisme en Verwante Stoornissenschaal voor Zwakzinnigen-Revisie (AVZ-R); revised assessment of autism and related disorders in ID [21]; AUTI-R; diagnostic schedule for the diagnosis of early developing autism in ID-revised version [22]; Vragenlijst voor Inventarisatie van Sociaal gedrag van Kinderen (VISK); interview for the inventory of social behaviour in kids [23]).

To compare data from 9 years before: FSIQ = 83; VIQ = 83; PIQ = 84 (WAIS). Current prescription: carbamazepine (as a mood stabilizer); oxazepam (if necessary) paroxetine; movicolon; carbasalaatcal-cium; levocabastine; fluticason inhaler. Neuroimaging: no other results available than X-ray of the skull: hyperostosis frontalis interna. Medical history: EEG: paroxysmal generalzsed epileptic findings during provocation (hyperventilation); constipation and vague abdominal complaints; orthopaedic footwear for ankle pain, double-sided lateral epicon-dylitis; ear correction after protruding ears; chronic low back pain due to mild scoliosis complicated by a double-sided spinal disc herniation, confirmed through MRI; deep vein thrombosis; dust allergy; several scars due to self-harm.

## Discussion

Why should there be another case report on psychiatric disorders in triple X syndrome? Until now the literature concerning triple X syndrome has focused on the ‘nature’ and the genetic disorder in children and has thereby neglected the ‘nurture’ and gene-environment interactions. Moreover, development in adults with triple X syndrome has never been described systematically. This study reports on two adult patients with triple X syndrome who were diagnosed with a variety of psychiatric disorders and challenging behaviour. As stated above the majority of girls and women with triple X syndrome develop without major developmental disabilities [7]. In both of these cases ASD, psychotic features, self-harm and suicidal behaviour and low self-esteem played a significant role. Both of them were sexually abused.

Both of the patients grew up in a family unaware of the special developmental needs of the girls. The level of functioning in daily activities decreased during adulthood.

Psychiatric aspects due to an extra X chromosome in women have been described in several studies. In a case control study of 22 women with triple X syndrome, Kidd et al. [24] demonstrated that sub-average intellectual functioning and psychotic disorders were common. Moreover, impairments in social and daily activities were considered to be serious:

All but one of the 14 triple X patients about whom these details were available were graded as having had ‘serious’ impairment of work efficiency and ‘serious’ difficulties in interpersonal relationships and social interaction during the year prior to admission (p. 91),

and

The individual clinical characteristics of the triple X patients were examined in detail against those of the control patients. The triple X patients differed significantly from the controls in showing a greater intensity of impairment of interpersonal relationships and a greater degree of social withdrawal. In addition, when compared with controls, a higher proportion of triple X patients than controls showed retardation of speech and action, ideas of reference and persecutory ideas’ (p. 91).

In two cases, suicidal impulses were reported [24]. Olanders [25] published the largest case series on 39 women with an extra X chromosome, including 24 girls and women with triple X syndrome, women with mosaic conditions and one 48,XXXX case. More than half of the cases were found in asylums, others in schools for special education, an epilepsy clinic, etc. [26]. In this biased population six women were of normal intelligence and 14 had mild ID or borderline intelligence [27]. Psychotic features were common, especially paranoid symptoms [28]. Autistic features were not mentioned. Six 47,XXX cases showed suicidal behaviour (in comparison, only one out of the 15 other cases) and two of them were sexually abused as a child or adolescent girl (in comparison, none of the other cases) [29]. Olanders [25–29] did not analyse occupational development and did not provide enough details to judge whether cases showed a decline in occupational development. Woodhouse et al. [30] reported on two cases with psychiatric disorders in triple X syndrome. Psychotic features with or without epilepsy and sub-average intelligence were described. In the first case they described the patient as interacting poorly with other children and having few friends at school.

Robinson et al. [7] summarized data on an unbiased population in a multicentre study:

Twenty-five (68%) reported no behaviour problems or concerns. The other 12 girls (11-24 years of age) were characterized with such diagnoses as mild depression, having a conduct disorder or being under-socialized. Several centres noted that concerns for interpersonal relationships were apparent. The girls were more vulnerable to a stressful home life than were their sibs or peers (p. 227).

Psychotic symptoms were not reported. Neither were suicidal behaviour and sexual abuse [7]. One of the centres in this study, Edinburgh, re-examined 16 of these girls during adulthood. Four of the 16 triple X girls were referred to a psychiatrist: one case of trichotillomania and three cases of behavioural disorders. Two of these four patients were diagnosed with a major depressive disorder. Two cases were diagnosed as cyclothymic personality and three for labile personality. One case suffered from non-psychotic ideas of reference and dissociative periods [31]. Three years later these women were re-examined. Increased prevalence rates of schizoty-pal symptoms were found: higher levels of social anxiety, suspiciousness, restricted emotion and impulsivity/non-conformity, irritability and anger. Irritability and anger were associated with lower FSIQ levels [32]. It is noteworthy to consider that in these unbiased studies there was a considerable difference in the parents’ knowledge of the cytoge-netic disorder. Some research groups initially failed to inform parents about the chromosomal condition of their child [33].

Sexual abuse is a complex environmental factor. Both patients A and B and several cases described in the literature above have been sexually abused [29]. This raises the question of whether women with triple X syndrome are more vulnerable than others. Adverse consequences of sexual abuse, such as depression and deliberate self-harm, have been described in a study on adult women with and without a confirmed history of sexual abuse [34]. Sexual abuse was associated with increased rates of mental illness and behavioural problems and with symptoms of post-traumatic stress. The more serious the abuse, the more severe the symptoms that were reported [34]. Moreover, low pre-exposure cognitive ability seems to be a risk factor for post-traumatic stress disorder (PTSD) [35].

Suicidal behaviour also is a complex phenomenon. It may be associated with affective disorders, sexual abuse, poor social support or being unmarried as a young woman. The strongest predictor of suicidal behaviour in young women is the existence of a physical disorder [36, 37]. Patients A and B suffer from physical disorders.

Patients A and B have been diagnosed in adulthood for their genetic condition. An unknown condition in a child may pose some form of adversity when growing up, especially in cases in which the condition is associated with subtle developmental difficulties. This lack of understanding by parents and teachers and by the girls and women themselves may yield chronic stress and may increase the risk of developing psychopathologic conditions. This should be taken into account in evaluating the results of studies in triple X syndrome.

Both cases demonstrate that ASD may be associated with triple X syndrome. During the diagnostic procedures the Autism Diagnostic Observation Schedule (ADOS) [38] and/or the Autism Diagnostic Interview, Revised (ADI-R) [39] were not available in Dutch. As there was no reasonable doubt about the psychiatric diagnosis, another assessment was judged as an unnecessary burden for these women. The literature revealed social impairments in triple X syndrome [7, 24]. MCDD is characterized by impairments in social interaction and social sensitivity, impairments in the regulation of affective state and impairments in the regulation of thought disorders. Patients with MCDD are less disturbed in social interaction but appear to be more impaired with regard to thought disorders, primitive anxieties and aggression [40]. This might explain the fact that in the study by Bishop et al. [9] no triple X girls were diagnosed with an ASD. However, in a large sample with ASD *(n* = 933) no sex chromosomal aneuploidies were detected [2]. The relation between triple X syndrome and ASD requires further study to learn another autism-lesson from the X chromosome [41].

ASD (including MCDD) have been found to be associated with psychotic disorders [42]. Patient A showed acoustic hallucinations and Patient B showed paranoid tendencies. Van Os et al. [43] recently demonstrated that psychotic syndromes (psychosis, motivational impairment, affective dysregulation and alterations in information processing) are highly heritable and associated with environmental factors such as early-life adversity. The unknown genetic disorder and the sexual abuse represent considerable developmental adversity that may partially explain the onset of a psychotic syndrome. Moreover, the psychotic syndrome is associated with motivational impairment and alterations in information processing, which might partially explain the decrease in daily-life functioning [43].

Both patients were able to hold a regular job after leaving school, but, more than two decades later, they require intensive support in activity centres. Kidd et al. [24] have already mentioned the serious impairment of work efficiency in triple X women with psychiatric disorders. There seems to be a decrease in the level of neurocognitive functioning, although the use of different versions of WAIS may to some extent explain the differences between the subsequent assessment results. Another explanation of the decline in functioning at work is that this decline is part of the behavioural phenotype of the triple X syndrome itself. Or the decline is partially explained by the psychotic disorder and partially by triple X syndrome itself. Moreover, PTSD may be associated with neurobiological changes [44] and neurodegenerative disorders [45]. So, gene-environment interactions may—partially—explain the decline in functioning.

## Conclusion

The presented cases clearly suffer from referral bias, but there are still some lessons to be learned from these cases in planning further research. The study of behavioural aspects in triple X syndrome requires a developmental perspective until late adulthood. Both cases illustrate that, in triple X syndrome, ASD and MCDD should be considered, especially when associated with ID. Girls and women with triple X syndrome may be more vulnerable to sexual abuse with a higher risk of subsequent mental health problems. Whether cognitive decline in adults with triple X syndrome is part of the syndrome or has to be explained through gene-environment interactions requires further longitudinal studies. The psychological consequences of an early vs a late genetic diagnosis in ID and/or psychiatric disease should be taken into account in evaluating the results of studies in triple X syndrome (and perhaps other genetic conditions). Because there are many unanswered questions in this relatively common disorder, further research is needed, especially in adults.
